# WearMoCap: multimodal pose tracking for ubiquitous robot control using a smartwatch

**DOI:** 10.3389/frobt.2024.1478016

**Published:** 2025-01-03

**Authors:** Fabian C. Weigend, Neelesh Kumar, Oya Aran, Heni Ben Amor

**Affiliations:** ^1^ Interactive Robotics Laboratory, School of Computing and Augmented Intelligence (SCAI), Arizona State University (ASU), Tempe, AZ, United States; ^2^ Corporate Functions-R&D, Procter and Gamble, Mason, OH, United States

**Keywords:** motion capture, human-robot interaction, teleoperation, smartwatch, wearables, drone control, IMU motion capture

## Abstract

We present WearMoCap, an open-source library to track the human pose from smartwatch sensor data and leveraging pose predictions for ubiquitous robot control. WearMoCap operates in three modes: 1) a Watch Only mode, which uses a smartwatch only, 2) a novel Upper Arm mode, which utilizes the smartphone strapped onto the upper arm and 3) a Pocket mode, which determines body orientation from a smartphone in any pocket. We evaluate all modes on large-scale datasets consisting of recordings from up to 8 human subjects using a range of consumer-grade devices. Further, we discuss real-robot applications of underlying works and evaluate WearMoCap in handover and teleoperation tasks, resulting in performances that are within 2 cm of the accuracy of the gold-standard motion capture system. Our Upper Arm mode provides the most accurate wrist position estimates with a Root Mean Squared prediction error of 6.79 cm. To evaluate WearMoCap in more scenarios and investigate strategies to mitigate sensor drift, we publish the WearMoCap system with thorough documentation as open source. The system is designed to foster future research in smartwatch-based motion capture for robotics applications where ubiquity matters. www.github.com/wearable-motion-capture.

## 1 Introduction

Tracking and estimating the human pose is essential for applications in teleoperation (Hauser et al., 2024), imitation learning (Fu et al., 2024), and human-robot collaboration (Robinson et al., 2023). To date, camera-based approaches are the gold standard for capturing human position and motion (Desmarais et al., 2021; Robinson et al., 2023). While purely optical motion capture solutions provide a high degree of accuracy, they are also subject to line-of-sight issues, which typically confines their use to controlled environments (Fu et al., 2024; Darvish et al., 2023). This requirement of controlled environments is even more prominent in human pose estimation advances in Virtual Reality (VR), and Mixed Reality methods (Walker et al., 2023), which typically require the user to wear VR headsets, or heavily rely on camera-based tracking.

The most prominent alternatives to optical solutions are based on Inertial Measurment Unit (IMU) sensors (Noh et al., 2024; Hindle et al., 2021). These methods employ customized IMU-based solutions (Prayudi and Kim, 2012; Beange et al., 2018; Li et al., 2021) on low-cost wearable embedded system (Raghavendra et al., 2017), possibly in fusion with optical methods for enhanceed accuracy (Malleson et al., 2017; Shin et al., 2023). Unlike optical methods, IMUs do not require a direct line of sight because they are directly attached to the user’s body. Commercial IMU motion capture systems incorporate up to 17 IMUs, enabling highly accurate non-optical human pose estimation (Roetenberg et al., 2009). Configurations with fewer sensors benefit from advances in deep-learning to obtain reliable lower-fidelity human poses (Huang et al., 2018). However, IMU-based motion capture systems typically require specialized IMU units and calibration procedures, thereby hindering their portability and applicability for inexperienced users (Huang et al., 2018; Roetenberg et al., 2009).

With the constantly growing popularity of consumer wearables, IMU-based motion capture from smartwatch and smartphone data offers perhaps the most ubiquitous solution (Lee and Joo, 2024). The recent IMUPoser (Mollyn et al., 2023) and SmartPoser (DeVrio et al., 2023) demonstrate that, even though consumer wearables motion capture may be less accurate than their optical and specialized IMU-based counterparts, these solutions are attractive because users tend to have these devices on them most of the time, enabling pose tracking at anytime and anywhere.

**FIGURE 1:**
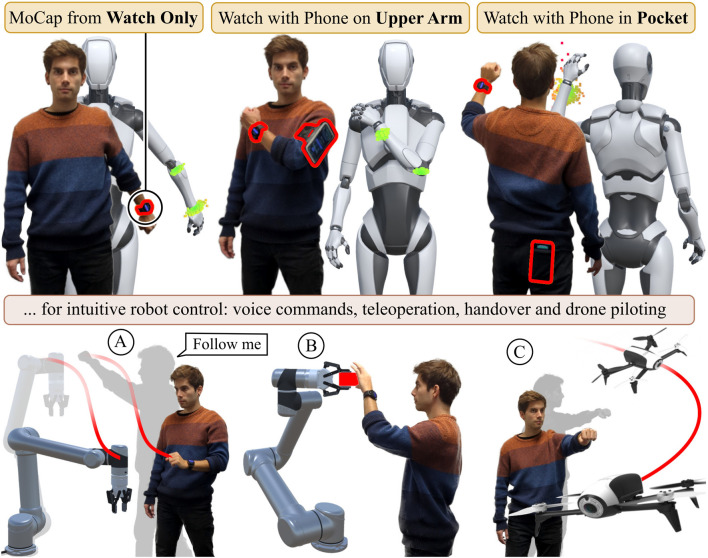
Our WearMoCap system features three modes: *Watch Only* requires a single smartwatch only. For *Upper Arm* we use a common fitness arm strap for the connected smartphone. The *Pocket* mode tracks the arm pose and uses the phone to determine changes in body orientation. We evaluate all modes in real-robot tasks, i.e., teleoperation, intervention **(A)**, handovers **(B)** and drone piloting **(C)**.

Despite these advances in ubiquitous pose tracking, smartwatch applications in robotics often merely utilize roll, pitch, yaw and gesture based control (Villani et al., 2020a), or on-body sensors for cognitive stress and alertness (Lee et al., 2015; Villani et al., 2020b). We have recently demonstrated the opportunities of motion capture from smartwatches for ubiquitous robot control (Weigend et al., 2023b; 2024). Under a fixed-body-orientation constraint, we showed that a single smartwatch facilitates teleoperation tasks (Weigend et al., 2023b). The additional sensor data from a smartphone in the pocket allows for tracking body orientation as well (Weigend et al., 2024; Weigend et al., 2023a). To foster future research in ubiquitous motion capture for robotics, in this work, we present WearMoCap—a comprehensive wearables-based motion capture system to unify and augment previous approaches in one system. As depicted in Figure 1, WearMoCap has three modes of operation for different levels of precision and portability. Improving on previous works, we benchmark WearMoCap extensively on three large-scale datasets, and show successful demonstration on multiple real-world robotics tasks.

We publish WearMoCap as an open-source library, together with extensive documentation, as well as all our training and test data. Specifically, our contributions are.

• We unify previous and new pose tracking modalities, visualizations, and robot interfaces in one system under the name WearMoCap.
• We introduce a more precise *Upper Arm* pose tracking mode using an off-the-shelf fitness strap.
• We evaluate each system modality on large-scale datasets from a range of consumer devices, up to 8 human subjects, and by comparing them in real-robot tasks.

Overall, we envisage this paper to be a streamlined framework for wearable motion capture with three modes, intended to facilitate data collection and future research into human-robot interaction through smartwatch and smartphone motion capture.

## 2 Methods

This section introduces the system architecture and operation. Section 2.1 covers system modules and formalizes the data flow. Section 2.2 describes calibration procedures, followed by the methodology for each pose prediction mode described in Section 2.3. Finally, Section 2.4 covers additional control modalities that we use for our evaluation on real-robot tasks. Each section defines our contributions and additions to the methodology previous works.

### 2.1 System overview and architecture

WearMoCap streams sensor data from smartwatches and phones, and computes pose estimates using them for robot control. As depicted in Figure 1, the system operates in three modes: 1) The *Watch Only* mode produces arm pose estimates using the sensor data of a single smartwatch. 2) The *Upper Arm* mode further employs a smartphone strapped to the upper arm. The combined sensor data of watch and phone allow for more precise arm pose estimates. 3) The *Pocket* mode requires the user to wear the watch on their wrist and place the phone in any of their pockets. This allows for tracking both the body orientation and arm pose. While the Watch Only mode is based on Weigend et al. (2023b) and the Pocket mode on Weigend et al. (2024), the Upper Arm mode is introduced by this paper.

**FIGURE 2:**
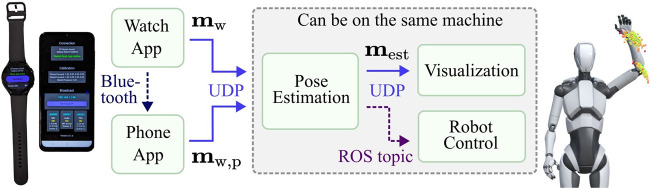
A schematic of the data streams between modules. The pipeline from smartwatch to visualization defaults to UDP. The pose estimation module can publish to the Robot Operating System (ROS).

WearMoCap unites all three modes in one framework. To ensure that users can deploy and switch between WearMoCap functionalities easily, we developed WearMoCap as a modular system (Figure 2). The system consists of the following components: i) apps to stream sensor data to a remote machine, ii) a pose estimation module to transform received sensor data into poses, iii) a visualization module that renders pose estimates and distributions using a 3D avatar, and iv) an interface to the Robot Operating System (ROS) for robot control. The apps are written in Kotlin and require Wear OS and Android OS. Pose estimation and the ROS interface are written in Python, and the visualization utilizes Unity3D and C# scripts. The communication between modules is facilitated using UDP messages. The only exceptions are robot control, which uses a ROS topic, and communication from the watch to the phone app, which is realized via Bluetooth.

The user initiates the data stream by pressing a button on the watch app. Messages from the watch app, $m_{\text{w}}$ , comprise:

$$m_{\text{w}}={\left[\Delta t_{\text{w}},t_{\text{w}},\theta_{\text{w}},\phi_{\text{w}},v_{\text{w}},\alpha_{\text{w}},\rho_{\text{w}},\gamma_{\text{w}},\theta_{\text{w,init}},\rho_{\text{init}}\right]}^{⊤},$$

with $m_{\text{w}}\in R^{27}$ . Δt is the time since the last message. The timestamp $t\in R^{4}$ contains the current hour, minute, second and nanosecond. The virtual rotation vector sensor θ by Android and Wear OS provides a global orientation quaternion $\theta \in R^{4}$ . Angular velocities are provided by the gyroscope $\phi \in R^{3}$ . Additionally, we integrate linear acceleration measurements $\alpha \in R^{3}$ over Δt to obtain velocities $v\in R^{3}$ . The value ρ is the atmospheric pressure sensor and the measurements $\gamma \in R^{3}$ are readings from the gravity sensor. The $\theta_{\text{w,init}}\in R^{4}$ and $\rho_{\text{init}}\in R$ are saved orientation and pressure readings from the calibration (Section 2.2).

In the Upper Arm and Pocket modes, the watch streams $m_{\text{w}}$ to the phone via Bluetooth. The phone then augments received messages with its own sensor data, and forwards the combined message $m_{\text{w,p}}$ to the host machine, where:

$$m_{\text{w,p}}={\left[m_{\text{w}}^{⊤},\Delta t_{\text{p}},t_{\text{p}},\theta_{\text{p}},\phi_{\text{p}},v_{\text{p}},\alpha_{\text{p}},\rho_{\text{p}},\gamma_{\text{p}},\theta_{\text{p,init}}\right]}^{⊤},$$

with $m_{\text{w,p}}\in R^{53}$ .

The pose estimation module receives $m_{\text{w,p}}$ or $m_{\text{w}}$ and computes pose estimates. To this end, it calibrates orientation values according to the procedure presented in Section 2.2 and makes predictions according to the corresponding mode methodology in Section 2.3. Then, it outputs a message summarizing the pose $m_{\text{est}}$ as

$$m_{\text{est}}={\left[\begin{array}{c} \underset{\text{hand}}{\underbrace{q_{\text{ha}},p_{\text{ha}}}},\underset{\text{lower arm}}{\underbrace{q_{\text{la}},p_{\text{la}}}},\underset{\text{upper arm}}{\underbrace{q_{\text{ua}},p_{\text{ua}}}},\underset{\text{hip}}{\underbrace{q_{\text{hi}}}} \end{array}\right]}^{⊤},$$

with $m_{\text{est}}\in R^{25}$ , quaternions $q\in R^{4}$ and origin positions $p\in R^{3}$ . The pose estimation module can either record $m_{\text{est}}$ to a file, send them to the visualization module, or, publish to a ROS topic for robot control.

**FIGURE 3:**
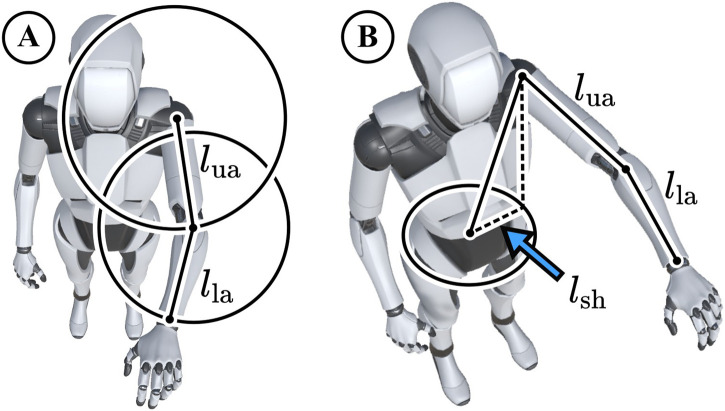
**(A)** Possible wrist and elbow positions around the shoulder lie on spheres with the radii of our standard upper arm length 26 cm $\left(l_{\text{ua}}\right)$ and lower arm length 22 cm $\left(l_{\text{la}}\right)$ . **(B)** We provide joint positions relative to the hip origin. If the forward-facing direction is not constrained, all possible shoulder positions lie on a circle. The shoulder offset $l_{\text{sh}}$ from the hip is X: -17.01 cm, Y: 43.1 c, Z: -0.67 cm.

The reference frame for all final positions is relative to the hip origin. For estimating joint positions through forward kinematics, we facilitate default arm lengths and shoulder offsets. As shown in Figure 3, the default left shoulder origin relative to the hip was set to X: -17.01 cm, Y: 43.1 cm, Z: -0.67 cm, which was determined as an average from our first three human subjects. Moreover, the default upper arm and lower arm lengths were set to 26 cm and 22 cm respectively. These settings worked well for all our experiments but developers can easily adjust the defaults in the bone_map.py script in our repository.

A local WiFi connection is sufficient to establish the connections between the devices, there is no requirement for internet connectivity. The device synchronization is maintained as follows: First, the watch sends its data to the phone, along with the associated timestamps. The phone maintains a queue to collect the timestamped data from the watch, and then collects its own sensor data at the fastest rate possible. Once the phone completes the collection of a new array of its sensor values, it processes the data in the queue from the watch. The phone integrates the watch data over time and aligns it with its own data. This way, the final output from the phone contains the most recent phone sensor data along with the integrated watch data, accurately matched to the corresponding time points.

### 2.2 Calibration

**FIGURE 4:**
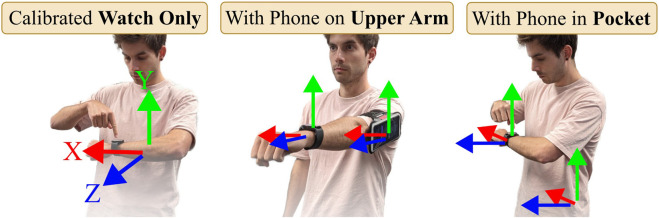
All modes start with a one-second calibration pose. For Watch Only and Pocket mode the user holds their arm parallel to the hip. In Upper Arm mode the user stretches their arm forward.

Motion capture requires a set of transformations to bring body joints and IMUs into the same reference frame. Traditionally, this involves calibration procedures like standing in a T-Pose (Roetenberg et al., 2009; Mollyn et al., 2023). We implement a seamless calibration pose for each mode, asking the user to hold a respective pose (as depicted in Figure 4) for one second.

For the Watch Only and Pocket modes, the user starts streaming with the watch app while holding their lower arm parallel to the chest and hip. The watch verifies this position using the gravity and magnetometer sensors. Then, it records the initial watch orientation sensor reading $\theta_{\text{w,init}}$ , such that the pose estimation from then on computes the calibrated orientation as

$$q_{\text{w,cal}}=\theta_{\text{w}}\cdot \theta_{\text{w,init}}^{-1}.$$

Further, the watch records the initial atmospheric pressure $\rho_{\text{init}}$ , so that we can compute the relative atmospheric pressure:

$$\rho_{\text{cal}}=\rho -\rho_{\text{init}}.$$

The calibration for the phone data operates similarly. In the Pocket mode, the phone orientation $q_{\text{p,cal}}$ is calibrated in the same way as the watch orientation $q_{\text{w,cal}}$ because the hip forward direction aligns with the watch forward direction (Figure 4 on the right). In the Upper Arm mode, the user stretches their arm forward to put the upper arm into a known position relative to the lower arm and hip (Figure 4 in the middle). Calibrating the phone orientation in this position allows aligning $q_{\text{p,cal}}$ with the upper arm orientation and hence remains unaffected by varying body proportions. Figure 4 depicts the result: In the start pose, the calibrated device orientations equate to identity quaternions, i.e., no rotation.

We describe the detail of the calibration process along with the average duration for each mode in the following subsection.

#### 2.2.1 Watch Only

The user has to hold the watch in a calibration pose as shown in Figure 4. The watch uses the gravity sensor to assess if it is positioned with its screen parallel to the ground. If the z-value of the gravity sensor is > 9.75 $\text{m}/{\text{s}}^{2}$ (perfect orientation would be the gravity constant 9.81 $\text{m}/{\text{s}}^{2}$ ), the watch indicates that it is ready to calibrate. The user can then initiate the calibration by triggering the start button. The app collects the watch orientation and atmospheric pressure sensor values for 100 ms and averages them. These measurements serve as the calibration values and future measurements are set relative to this initial average. Therefore, the calibration procedure requires the user to bring the watch into the correct position and collects 100 ms of data. The procedure is typically finished in 1 s.

#### 2.2.2 Upper Arm

For this calibration procedure, the user has to complete two steps. Both are depicted in Figure 4. Step 1 is the same as Watch Only: If the z-value of the gravity sensor is > 9.75 $\text{m}/{\text{s}}^{2}$ , the watch indicates that it is ready to calibrate. Upon button trigger, the app collects 100 ms of orientation measurements and saves the average as the initial pose orientation. Subsequently, the watch vibrates to signal the user to stretch their arm forward. The watch then keeps track of orientation changes. As soon as the *z*-axis of the gravity sensor is > 9.75 $\text{m}/{\text{s}}^{2}$ again and the global y-orientation changed by more than 80° , the watch sends a message to the phone. Upon receiving the message, the phone collects its own global orientation for 1,000 ms. The average is the phone orientation calibration and future orientations are estimated relative to the calibration value. Altogether, the user has to stand in two poses and the devices collect data for 1,100 ms. The procedure is typically finished in about 2–3 s.

#### 2.2.3 Pocket

The user places the smartphone in their pocket. The user holds the watch in front of their body as shown in Figure 4. Once the z-value of the gravity sensor is > 9.75 $\text{m}/{\text{s}}^{2}$ , the watch indicates that it is ready to calibrate. The watch collects orientation and pressure for 100 ms, then immediately sends a message to the phone, and the phone records its own orientation for 100 ms. Recorded orientations serve as calibration measures. Typically, this procedure is completed within 2 s.

### 2.3 Pose estimation in motion capture modes

**FIGURE 5:**
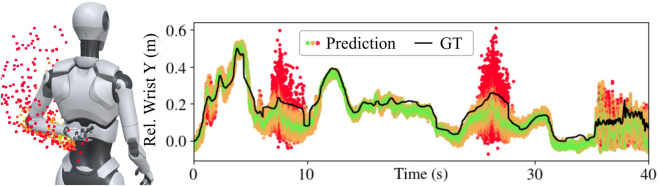
Stochastic forward passes produce ensembles of possible arm poses. Individual predicted wrist positions are shown as dots, colored based on their distance from the ensemble mean—green indicates closer proximity to the mean, while red signifies greater deviation. High variance within the ensemble reflects high uncertainty, which might occur in unergonomic poses or during rapid movements. The true wrist position is indicated as ground truth (GT).

This section outlines the pose estimation methodology for the three motion capture modes. All three modes employ neural network-based approaches with stochastic forward passes to obtain a distribution of solutions Gal and Ghahramani (2016). In Figure 5, possible solutions are depicted as small cubes colored according to their distance from the mean. Wide distributions are indicative of unergonomic arm poses or fast jittering motions.

#### 2.3.1 Watch only

For the Watch Only mode, we employ the derived optimal neural network architecture from Weigend et al. (2023b). An LSTM estimates the lower arm orientation $q_{\text{la}}$ and upper arm orientation $q_{\text{ua}}$ from a sequence of watch sensor data $m_{\text{w}}$ with calibrated orientation and pressure. The output message $m_{\text{est}}$ sets the estimated hand orientation $q_{\text{ha}}$ equal to $q_{\text{la}}$ , and subsequently, we derive positional values through forward kinematics by assuming an approximate lower arm length of 22 cm and upper arm length of 26 cm. The Watch Only mode requires a constant forward-facing direction, i.e., the hip orientation estimate $q_{\text{hi}}$ is constant and arm pose tracking is stable as long as the user does not change their forward-facing direction after calibration. While the general inputs and targets are the same as in Weigend et al. (2023b), we use slightly altered hyperparameters: Our LSTM has 2 hidden layers with 256 neurons each and we use a sequence length of 12.

#### 2.3.2 Upper arm

The previous Watch Only mode infers the upper arm orientation from the smartwatch sensor data only. This is sparse data for arm pose predictions. Therefore, we now introduce the additional Upper Arm mode, which facilitates more sensor data to infer the entire arm pose by placing the smartphone directly on the upper arm. As described earlier, the user can use an off the shelf fitness strap. We use an LSTM to predict $q_{\text{la}}$ and $q_{\text{ua}}$ from the last four combined watch and phone sensor data $m_{\text{w,p}}$ readings. Similar to the Watch Only mode, we estimate positions through forward kinematics with default arm lengths of 22 cm for the lower arm and upper arm length of 26 cm. We determined our hyperparameters through gridsearch. The best result was achieved with with three LSTM layers of 128 neurons applying a dropout of 0.2 on the last one. Further, a sequence length of 4, batch size of 32 and learning rate of 0.0015 lead to the best results. Our loss function was the L1 loss and we used the Adam optimizer.

With this mode, after calibration, the user is free to turn around. However, this mode does not provide body-orientation estimates, which means the lower and upper arm orientations $q_{\text{la}}$ and $q_{\text{ua}}$ capture the correct arm pose in any forward-facing direction but the hip orientation estimate $q_{\text{hi}}$ is constant.

#### 2.3.3 Pocket

This mode is based on Weigend et al. (2024) and uses a Differentiable Ensemble Kalman Filter to update an ensemble of states from previous estimates and the watch and phone sensor data $m_{\text{w,p}}$ . Each ensemble member describes the orientation of the lower arm $q_{\text{la}}$ , upper arm $q_{\text{ua}}$ , and the rotation around the up-axis of the hip $q_{\text{hi}}$ . This allows us to compile the pose estimation $m_{\text{est}}$ and determine joint positions $p_{\text{ha}}$ , $q_{\text{la}}$ , $q_{\text{ua}}$ through forward kinematics. We retained the hyperparameter settings of Weigend et al. (2024) but trained the filter anew on the larger dataset that we compiled for this work.

### 2.4 Additional control modalities

**FIGURE 7:**
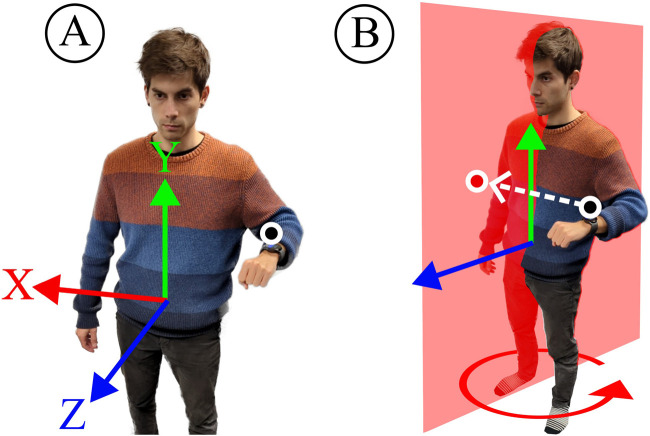
We use two control modalities to determine end-effector positions. Modality **(A)** leverages forward kinematics with default arm lengths to return the wrist origin relative to the hip. Modality **(B)** estimates the wrist origin projected onto the sagittal plane.

For teleoperation tasks that involve advanced gripper control (see Section 3.4), we stream microphone data to issue voice commands. This is done by transcribing the recorded audio signal into voice commands utilizing the Google Cloud speech-to-text service^1^. We also implement two positional control modalities (A and B in Figure 7). Voice commands were used in our previous works (Weigend et al., 2023b; 2024) and Modality A was utilized in Weigend et al. (2023b), while Modality B was proposed in Weigend et al. (2024). Typically, users expect to control the robot with their hand position. Therefore, both of our control modalities translate wrist/hand positions into control commands, e. g., end-effector positions.

With Modality A, we determine the wrist position relative to the hip origin. This is then directly translated to the end-effector position relative to its base. Modality B requires the dynamic hip orientation estimates $q_{\text{hi}}$ in Pocket mode. Here, the local forward direction (Z) aligns with the sagittal plane (red) given by the current hip orientation. The projected wrist coordinates define the end-effector position on that plane.

The main difference between Modality A and B is the reduction in interacting degrees of freedom to reduce potential compounding errors. With Modality A, the end-effector X-position is determined by the complete kinematic chain $q_{\text{hi}}$ , $q_{\text{ua}}$ , and then $q_{\text{la}}$ . In contrast, Modality B determines the target X-position through the hip orientation $q_{\text{hi}}$ and then the projected distance and elevation of the wrist. This reduces potential compounding errors but makes it more difficult to adjust the X-position without affecting Y and Z-positions. Therefore, Modality B is more suitable for circular control motions with the user at the center. On the other hand, Modality A is more suitable for situations where the user has a more constant forward facing direction. The evaluation of both control modalities on real-robot tasks is discussed in Section 3.4.

## 3 Results

We evaluate the performance of WearMoCap in real-robot tasks and on large-scale datasets from multiple studies and across multiple devices (smartwatches and smartphones). The first Section 3.1 covers the composition of our training and test datasets. Section 3.2 details prediction performance on our test datasets and compares it to related work; Followed by Section 3.4, which describes the evaluation on four real-robot tasks and concludes by summarizing results and limitations.

### 3.1 Composition of datasets

We composed a large-scale dataset by merging datasets collected from previous studies (Weigend et al., 2023b; 2024), and augmenting them with data collected for this study. We employed the following devices for data collection: smartwatches—Fossil Gen 6 Men’s, and Samsung Galaxy Watch 5 40 mm version (RM900) and 45 mm version (RM910); smartphones—OnePlus N100, TCL 40XL and Samsung Galaxy A23G. Out of these, only Samsung Galaxy A23G and Samsung Galaxy Watch 5 were used in the datasets from previous studies (Weigend et al., 2023b; Weigend et al., 2024). The rest are new to this study. The OS version on the Samsung Watches was WearOS 4 which is based on Android 13. The Fossil Gen 6 had WearOS3 based on Android 11. The sampling frequency of newer phones such as Samsung A23 is 90 Hz, while phone such as OnePlus N100 transmit data at 60 Hz sampling frequency. Since our model input includes delta time, the model is able to account for fluctuations and differences in frequency. For all previous and new datasets, the ground truth was obtained with the optical motion capture system OptiTrack (Nagymáté and Kiss, 2018). The OptiTrack motion capture environment featured 12 cameras, which were calibrated before data collection. Human subjects wore a 25-marker-upper-body suit along with the smartwatch on their left wrist and phone on upper arm or in pocket. We collected lower arm, upper arm, and hip orientations with time stamps. The system recorded poses at 120 Hz. In post processing, we matched WearMoCap data with the OptiTrack pose closest in time. All human subjects (8 Males; Mean age: 25 ± 3) provided written informed consent approved by the institutional review board (IRB) of ASU under the ID STUDY00017558. The recruitment criteria for the subjects were as outlined in the IRB: English-speaking adults between the ages of 18 and 70 with no current physical impairments that affect arm or body movements.

To collect data for the Watch Only mode, we asked subjects to perform single-arm movements under a constant forward-facing constraint. We combined this data with data from Weigend et al. (2023b), which resulted in a dataset with 0.6 M observations. Here, each observation refers to a collected data row.

For the Upper Arm mode, we asked 5 subjects to perform similar movements as above, but with a phone strapped on to their upper arm. For the Upper Arm mode, we did not enforce a constant forward direction. Additionally, subjects were encouraged to occasionally perform teleoperation-typical motions, such as moving the wrist slowly in a straight line. We showed demonstrations of writing English letters on an imaginary plane as examples of such motions. However, subjects were not strictly instructed to perform these movements and some chose not to or forgot. Therefore, not all recordings contained these teleoperation-typical movements. This resulted in a dataset with 0.4 M observations.

For the Pocket mode, subjects had to keep a smartphone in any of their pockets. For data collection, subjects were free to move their arm in any direction and without the forward-facing constraint. Further, the pose estimation in Pocket mode only requires the orientation sensor data $\theta_{\text{p}}$ of the phone (Weigend et al., 2024). This allowed us to retrospectively simulate phone-in-pocket data for collected Watch Only and Upper Arm data using the ground truth hip orientation $q_{\text{hi}}$ as an approximate calibrated phone orientation. All data combined compiled a dataset of 0.9 M observations.

Both the Upper Arm and Pocket modes do not restrict body orientation, which allowed us to augment the data. This was done by rotating $q_{\text{la}},q_{\text{ua}},q_{\text{hi}}$ as well as $q_{\text{w,cal}}$ and $q_{\text{p,cal}}$ around the global *Y*-axis. The global rotation is possible because all other sensor readings in $m_{\text{w,p}}$ are in the local device reference frame and, therefore, unaffected by changes in global Y-axis-rotation. We augment the data for the Upper Arm and Pocket modes two times by rotating around a random Y-angle. The dataset composition details for each mode are summarized in Table 1.

**TABLE 1:** Compiled dataset attributes for each WearMoCap mode.

Mode	Data	Augm.	#Subj.	Devices
Watch Only	0.6 M	-	7	3
Upper Arm	0.4 M	1.2 M	5	3×3
Pocket	0.9 M	2.6 M	8	3×3

The column Augm. indicates the dataset volume post augmentation, #Subj. indicates the number of subjects data was collected from, and Devices indicates the number of distinct devices data was collected with. 3×3 stands for three smartwatches and three smartphones.

**FIGURE 8:**
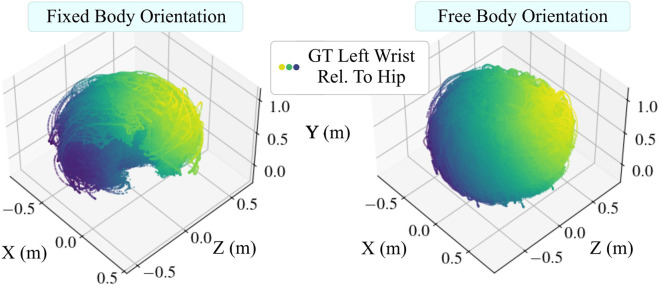
Using wrist positions as an example, this figure shows that our collected data covers the space of possible arm poses (position space). Dots are data points representing wrist positions relative to the hip obtained from the motion capture system, which also show differing arm lengths. Data points are colored according to the sum of their coordinate magnitudes. Left: Data points collected under the fixed forward-facing constraint. Right: Wrist positions collected without the fixed body orientation constraint form a full sphere.

For all the datasets, we provided the subjects with verbal instructions and brief demonstrations of motions that covered the position space well, and asked the subjects to perform them. We confirmed the variability of their motions by inspecting the 3D plots of their movement trajectories, which revealed that the data covers the position space. An example overview of all participant’s combined wrist positions is depicted in Figure 8. Our training and test data includes recording sessions of up to 10 min duration. The mean duration and other statistics such as number of sessions, average number of observations, etc. can be found in Table 2. Five of the subjects that were used to collect data in previous studies Weigend et al. (2023b), Weigend et al. (2024) were used again to collect new data in this study.

**TABLE 2:** Statistics of dataset incorporated from previous works, and additional data collected in this work.

Data source	Mean time (s)	Sum time (s).	Mean # obs.	Sum # obs.	# Sessions	# Subj.
Weigend et al. (2023b)	227±47	3,855	17±3 k	287 k	17	6
Weigend et al. (2024)*	500±100	5,501	17±3 k	185 k	11	4
(New) Cnst. body orient.**	409±116	5,323	24±6 k	305 k	13	5
(New) Free body orient.***	378±76	1,515	26±10 k	103 k	4	3

The first two rows represent previous studies. The bottom two rows represent new data collected in this study where subjects were asked to perform movements with constant forward-facing body orientation (Cnst. body orient.) and with free body orientation (Free body orient.). The asterisks indicate the modes for which the data was utilized (* Pocket Mode only; ** All modes; *** Upper arm and Pocket Mode).

### 3.2 Model accuracy

**FIGURE 6:**
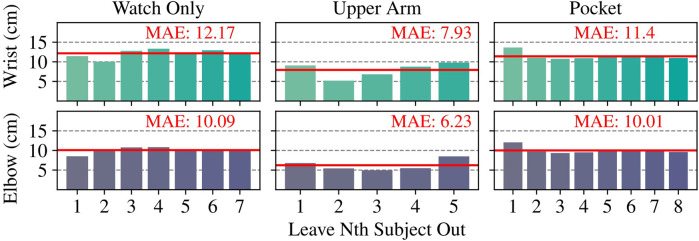
Euclidean Mean Absolute Error (MAE) of wrist and elbow position estimates in leave-one-subject-out cross validations. Specifically, we trained on data from all the subjects except the Nth subject, and tested on the Nth subject.

We employed our dataset to assess WearMoCap performance in two ways: all-subjects validation and leave-one-out validation. For the all-subjects validation, we utilized 3/4th of each subject’s data for training, reserving the remaining portion for testing. We train five models with randomly initialized weights and report the average error. We consider these results to be indicative of performance within controlled settings where the model can be fine-tuned on a known population. In contrast, the leave-one-out validation involves a cross-validation approach, where we systematically reserved all the data from one subject at a time for testing while training the model on the data from the remaining subjects. The leave-one-out performance measures the ability of the model to generalize to new subjects and is, hence more suitable to assess performance in real-world applications. Our results are summarized in Figure 6 and in Table 3 we compare against the state-of-the-art baseline methods wherever applicable.

**TABLE 3:** Model performance for each WearMoCap mode and comparison to baselines.

Watch Only baseline	Evaluation	Metric	Wrist (cm)		Elbow (cm)		Hip (°)
			Theirs	Ours	Theirs	Ours	Ours
Liu et al. (2023)	All	MAE	10.93	10.82±0.04	-	9.45±0.08	-
Wei et al. (2021).A	1out	MAE	8.5	12.17±1.03	8.5	10.09±0.73	-
Wei et al. (2021).B	1out	MAE	15	12.17±1.03	11.5	10.09±0.73	-
Upper Arm							
Joukov et al. (2017)	All	RMSE	6.9±2.7	6.79±0.57	5.2±2.6	4.24±0.31	-
Pocket							
DeVrio et al. (2023)	1out	MAE	15.1±1.42	11.4±0.87	10.0±0.9	10.01±0.81	4.17±0.5

The by the baseline chosen type of evaluation is characterized the by the Evaluations and Metrics columns. Abbreviations stand for: trained on data from all subjects (All), leave-one-out (1out), Mean Absolute Error (MAE), and Root Mean Squared Error (RMSE). We reported standard deviations where available.

#### 3.2.1 Watch only

As depicted in Figure 6 (Left), we trained seven distinct models for the Watch Only leave-one-out validation corresponding to seven different subjects. On average, the predicted wrist positions deviated by 12.17±1.03 cm and elbow positions by 10.09±0.73 cm. In the all-subjects validation, our model achieved slightly better prediction errors with 10.82±0.04 cm for wrist and 9.45±0.08 cm for elbow positions. In Table 3, we show that these results do not deviate strongly the works of Wei et al. (2021); Liu et al. (2023), which also estimated the arm pose from a single smartwatch on the wrist. The authors of Liu et al. (2023) evaluated their method using data from all subjects in the training and test set. Their method is able to estimate the wrist position in any forward-facing direction; however, they require inference in the same environment where the training data was collected. In our work, we enforce a constant forward-facing direction but allow for inference to be performed anywhere. The authors of Wei et al. (2021) evaluated their method using leave-one-out validation against two ground truth measures–the first using two IMUs (denoted as Wei et al. (2021). A in Table 3) and the second from a Kinect sensor (Wei et al. (2021). B). Their approach, akin to our Watch Only Mode, necessitates users to maintain a constant forward-facing direction. Our leave-one-out prediction error falls between the reported errors of Wei et al. (2021). A and Wei et al. (2021).B.

#### 3.2.2 Upper arm

Similar to our Upper Arm mode, Joukov et al. (2017) proposes the use of one IMU on the lower arm and the second on the upper arm. Their evaluation is based on all-subjects validation and uses RMSE as the performance measure. Table 3 shows that our errors of 6.79±0.57 cm for wrist and 4.24±0.31 cm for elbow positions are similar to those reported by Joukov et al. (2017), despite our mode being evaluated across multiple commercial devices and a wider range of motions. Figure 6 summarizes our leave-one-out validation results, where prediction errors were slightly higher with MAEs of 7.93±1.68 cm for wrist and 6.23±1.28 cm for elbow positions.

#### 3.2.3 Pocket

Similar to our Pocket mode, the authors of DeVrio et al. (2023) also leveraged data from a smartwatch and additional sensor data from a smartphone placed in the pocket. The authors conducted a leave-one-out evaluation. A comparison of WearMoCap to their reported results is shown in Table 3, and also here are comparable. With an average wrist error of 11.4±0.87 cm, WearMoCap appears to be more accurate for the wrist on our data, but marginally less accurate for the elbow with an error of 10.01±0.81 cm. Further, our method provides an additional hip orientation estimate with an average error of 4.17±05° .

All discussed methods are real-time capable. Our most computationally demanding mode is the Pocket mode, which achieves inference speeds of ∼ 62 Hz on a system equipped with an Intel^®^ Xeon(R) W-2125 CPU and NVIDIA GeForce RTX 2080 Ti.

### 3.3 Sensitivity analysis

To determine the relative importance of each input feature to our models, we conducted a sensitivity analysis where we left each sensor out, one at a time, in the Watch-Only mode. We noted effect on the model performance for prediction of Hand and Elbow positions in Table 4. The results show that leaving out the global orientation harms the performance the most, followed by gyroscope and accelerometer. While leaving out the atmospheric pressure sensor did not affect the accuracy significantly, we retained the sensor in our data.

**TABLE 4:** Model performance after removing individual sensors for sensitivity analysis.

Prediction	All	No gyro	No acc (vel, grav)	No orientation	No pressure
Hand	10.82 ± 0.04	11.06 ± 0.13	11.30 ± 0.10	19.26 ± 0.13	10.76 ± 0.09
Elbow	9.19 ± 0.08	9.45 ± 0.08	9.53 ± 0.07	12.41 ± 0.06	9.17 ± 0.06

For every condition, we trained 5 networks with randomly initialized weights, utilizing 75% of the data of every participant for training and 25% for testing. All numbers are in cm and are averaged over the 5 random networks. Results are shown for Watch Only mode.

### 3.4 Real-robot tasks

To assess the practical use of WearMoCap in robotics, we evaluate its application in four human-robot experiments, namely, Handover, Intervention, Teleoperation, and Drone Piloting tasks. The Handover and Intervention tasks were conducted for this work under the ASU IRB ID STUDY00018521. The Teleoperation and Drone Piloting tasks were conducted in Weigend et al. (2024) under the ASU IRB ID STUDY00018450. We picked these tasks such that our evaluation covers the three WearMoCap pose tracking modes Watch Only, Upper Arm, Pocket and control Modalities A and B with at least two experiments each. Section 3.4.5 discusses the results and compares them to the user performance with the OptiTrack system where possible. OptiTrack provides sub-millimeter accurate tracking and is therefore utilized as our state-of-the-art baseline (Nagymáté and Kiss, 2018; Topley and Richards, 2020). All human subjects (9 Males; 1 Female; Mean age: 25 ± 3) provided written consent. 4 human subjects performed all the robotic tasks, 1 subject performed teleoperation and drone tasks, 1 subject performed drone and intervention tasks, and the remaining performed only the drone task. While one subject had prior experience with drone piloting, none of the other subjects had any prior experience with any robotic tasks.

#### 3.4.1 Handover

In the Handover Task, an arm robot picks up an object from the table and hands it over to a human subject at a given location. Subjects sat on a rotating chair at a fixed location in front of a Universal Robot 5 (UR5). To do this task successfully, the robot must correctly track the human hand position. To this end, we provide the robot with the relative chair position, approximate sitting height, and arm lengths, such that it can estimate handover positions relative to its base.

**FIGURE 9:**
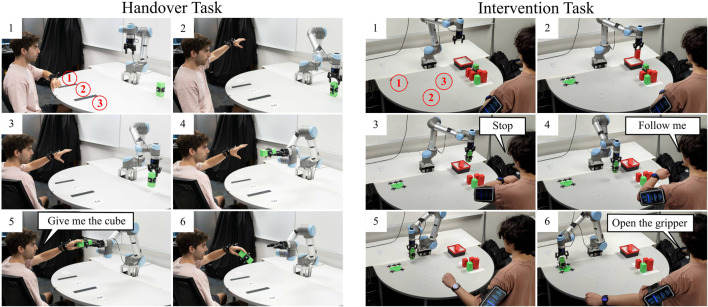
Handover Task: Human subjects used WearMoCap to perform handovers with a UR5. A voice command completed the handover and the robot let go of the cube. Intervention Task: The UR5 sorted the green and red cubes into the bin. Human subjects use WearMoCap to interrupt the robot and to place the green cube at a target location.

As depicted in Step 1 on the left of Figure 9, the tabletop area between the robot and the subject was divided into three areas. We ask subjects to perform handovers in each of these areas to ensure a range of diverse poses. With the subject’s hand in one of these areas, the subject performed two handovers—once with the hand at a low height and once with the hand at a higher height. The subjects then repeated this task for all the other areas at random. The subject’s orientation was fixed for Watch Only mode, but for the other two modes, they could change their orientation by rotating the chair.

Figure 9 summarizes the steps for each handover task. From the initial setup (Step 1), the subject raised their arm in one of the three locations at random (Step 2). Then, the robot picked up the green cube (Step 3). Given the known chair position and subject’s sitting height, we tracked the hand position of the subject using WearMoCap. The robot moved the cube toward the tracked hand position (Step 4). The subject then issued a voice command (Step 5) after which the robot released the cube (Step 6). Depending upon the accuracy of hand tracking, the subject had to move their hand by a certain “handover distance” to grab the cube.

Four human subjects performed 24 tasks each, comprising six handovers with Watch Only, Upper Arm, Pocket modes and with OptiTrack. We randomized the order of tracking modes to eliminate potential biases or learning effects. We computed the handover distance, which is the difference between the hand position and the cube at the time the participant triggered the voice command (Step 5). To compute the handover distance, we located the center of the user’s wrist and the center of the cube using Optitrack markers on both. Then we took the euclidean difference between the two. We also computed the handover time, which is the time that it takes for the robot to move toward the hand and complete the handover task (from Step 2 to Step 5).

#### 3.4.2 Intervention

In the Intervention Task, the human subject interrupts the robot during its routine when it makes a mistake, and performs corrective action. For this task, a UR5 robot was supposed to autonomously pick up a colored cube (green or red) and drop it at target locations of the same color. However, the robot was not trained to correctly place green cubes. As depicted in Step 1 on the right of Figure 9, the human subject stood in front of the robot and there were three possible target locations for the green cube. Whenever the robot picked up a green cube, the subject stopped the robot with a voice command and made it place the cube at the correct location.

Figure 9 summarizes the steps. The subject watched the robot (Step 2) and stopped it with a voice command from dropping a green cube at the red location (Step 3). Then, the subject instructed the robot to mirror their arm motion, i.e., move the robot end-effector in the same way as the subject’s wrist movement (Step 4). The WearMoCap algorithm, in conjunction with control Modality A (Figure 7), tracked the hand position and converted it into end-effector coordinates to control the robot (Step 5). The subject then issued another voice command (“Open the gripper”) to complete placing the cube at the correct location (Step 6).

Five subjects performed this task for each of the three green target locations and with each WearMoCap mode at random. The performance was evaluated with respect to the placement distance, which is the distance between the position of the placed green cube and the center of the target location. This was measured using OptiTrack. We also computed the task completion time, which is the time that elapsed between issuing the “Follow me” command and the “Open the gripper” command.

#### 3.4.3 Teleoperation

**FIGURE 10:**
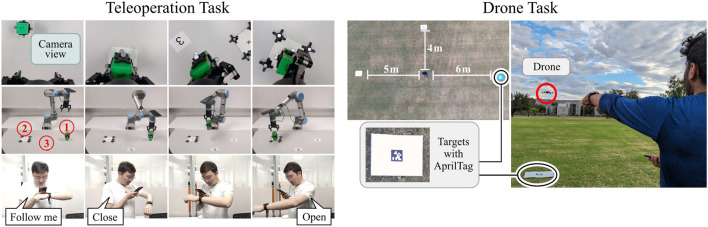
Teleoperation Task: Human subjects used WearMoCap to pick and place a cube. They were entirely removed from the robot and only watched the end-effector through a camera feed on the phone. Drone Piloting Task: Human subjects piloted a drone to three target locations in random order. Each location was marked with an AprilTag. If a tag was recognized through the drone camera feed the target was reached.

As depicted on the left in Figure 10, subjects controlled a UR5 to pick and place cubes from a remote location through a live camera feed on their smartphone. This was done as follows: the subject initiated the task with a “Follow me” voice command, which started the hand tracking. The subject maneuvered the robot end-effector toward the cube to be picked up. The subject then issued a “Close” voice command to grab the cube. Then, the subject maneuvered the robot end-effector to the target location and dropped the cube with “Open” voice command. We employed the Pocket mode of WearMoCap, in conjunction with control Modality B (Figure 7), to estimate the end-effector position for robot control. This combination allowed the subject to control the robot through changes in their body orientation, i.e., the robot turned left (right) whenever the subject turned left (right).

This task was performed by five subjects for six different configurations of pick-up and target locations of the cube. For one instance using OptiTrack and two instances using WearMoCap, the task execution failed because the subject knocked over the cube. For all successful completions of the tasks, we computed the placement accuracy, which is the distance between the placed cube and the target location, as measured by OptiTrack. We also computed the task completion time, which is the time elapsed between issuing the “Follow me” and “Open” commands.

#### 3.4.4 Drone piloting

In this task, subjects used motion capture to fly a commercial Parrot Bebop 2 drone to three target locations. Drone control via traditional remotes is hard to master while control through body motions can be more intuitive for inexperienced pilots Macchini et al. (2020). As shown on the right in Figure 10, with the subject at the center of a field, the three target locations were at distances of 4 m, 5 m, and 6 m in three directions. The targets were colored cardboard sheets with AprilTags (Wang and Olson, 2016). Subjects were instructed to fly the drone above these targets in a randomized order. A target was considered to be reached when its corresponding AprilTag ID was recognized through the downward-facing drone camera. The subjects controlled the drone with WearMoCap in Pocket mode, utilizing control Modality B. The drone used GPS and internal IMUs to follow the control commands in a stable trajectory.

Ten human subjects performed the task two times each: first, with WearMoCap and then with the original remote called SkyController. The performance was measured using drone piloting time which is the time it took for the drone from reaching the first target until reaching the third target.

#### 3.4.5 Results summary

We summarize the objective task metrics in Table 5. For each task, we compared the performance of WearMoCap against the baseline control methods.

**TABLE 5:** Summarized robot tasks results.

Task	Method	Dist. (cm)	Time (s)	Trials	Modality
Handover	OptiTrack	6.8 ± 1.6	9.2 ± 3.2	24	A
	Watch Only	+4.5 ± 9.7	+3.3 ± 8.1	24	A
	Pocket	+2.2 ± 3.6	+3.4 ± 6.3	24	A
	Upper Arm	+1.9 ± 3.7	+0.5 ± 5.2	24	A
Intervent.	OptiTrack	2.4 ± 1.5	17.5 ± 4.5	15	A
	Watch Only	+5.2 ± 6.0	+10.5 ± 7.8	15	A
	Pocket	+2.9 ± 4.3	+11.4 ± 10.7	15	A
	Upper Arm	+1.7 ± 5.2	+4.0 ± 5.6	15	A
Tele.	OptiTrack	4.5 ± 2.9	59.8 ± 16.5	29	B
	Pocket	+1.8 ± 6.7	+13.6 ± 28.9	28	B
Drone	SkyController	-	59.7 ± 27.8	10	B
	Pocket	-	- 19.2 ± 24.16	10	B

Distance errors and time differences are denoted in relation to the baseline. For example, the handover distance in Watch Only mode was on average +4.5±9.7 cm larger than when performing the same task with OptiTrack for motion capture. The Modality column indicates the utilized control modality from Figure 7.

The Handover and Intervention tasks investigate all WearMoCap pose estimation modes Watch Only, Upper Arm, and Pocket when using control Modality A and compare to OptiTrack as the baseline method. Expectedly, the Watch Only mode is more error-prone than its counterparts, evidenced by its higher handover distance (+4.5 cm) and intervention placement distance (+5.2 cm). The Upper Arm mode is the most accurate with an increase below +2 cm in both tasks. These results are consistent with the evaluation on test data in Section 3.2. It is also noteworthy that the Pocket mode too outperformed Watch Only mode in our distance metric. This is because it offers an additional degree of freedom to fine-tune positioning. However, due to this additional degree of freedom, the Pocket mode also incurred longer task completion times, because subjects had to balance changes in arm motion with changes in body orientation.

The Teleoperation and Drone tasks applied control Modality B, which relies on body orientation estimates in Pocket mode. Pocket mode with Modality B was highly accurate in terms of distance metric, with an increase of only 1.8±6.7 cm from the baseline OptiTrack for teleoperation. As in previous tasks, control through body orientation caused an increase in the completion times when compared to OptiTrack. However, when comparing to the SkyController remote control operation with non-expert drone pilots, WearMoCap incurred significantly shorter task completion times ( 19.2±24.16 s). This finding is limited to our specific drone task but still complements the finding of Macchini et al. (2020) that motion capture control can be more intuitive for inexperienced pilots.

## 4 Discussion

Reflecting on our presented results, this section discusses WearMoCap in detail: Section 4.1 contrasts all three WearMoCap modalities with their benefits and limitations. Section 4.2 discusses the broader significance of our framework, its limitations, and future work. Section 4.3 concludes this paper.

### 4.1 Modality trade-offs

Given the observed differences in model accuracy on test data, and varying real-robot task performance for each WearMoCap mode, we discuss the following trade-offs for their application.

#### 4.1.1 Watch only

Using only a smartwatch is the most convenient in terms of availability and setup, but the real-robot task results demonstrate a considerable increase in placement deviations and completion times in contrast to other modes. The applicability of the Watch Only mode depends on the task. If the application requires high-fidelity teleoperation control to perform pick-and-place tasks, the prediction deviations of about 10 cm are too large to be practical. Even though users were able to complete the Intervention task in Watch Only mode, the teleoperation required patience and users were not in full control. On the contrary, in a handover task, the human can compensate for the final centimeters by reaching. In such lower-fidelity applications, being able to replace an optical motion capture system with a single smartwatch is promising for future work.

#### 4.1.2 Upper arm

While an upper-arm fitness strap is widely used and available, it adds an extra step compared to the other two modes. Nevertheless, the increase in accuracy of arm pose tracking with two IMUs has previously been assessed in Yang et al. (2016); Joukov et al. (2017), and is confirmed by our results. Out of all WearMoCap modes, the Upper Arm mode is the most accurate on the test data and incurs the smallest deviations in our real-robot task completion times and placement accuracy compared to baselines. The relatively small placement deviations of below 2 cm suggest that this mode can be a viable alternative to robot control through motion capture from OptiTrack or Virtual Reality hardware when ease-of-setup is a concern and ubiquity matters.

#### 4.1.3 Pocket

The Pocket mode allows for the most seamless experience because users simply put the phone in their pocket and are free to turn their body. This is in contrast to the Watch Only mode, where users have to maintain a constant forward-facing direction. Our Handover and Intervention real-robot tasks indicate that the additional tracking of body orientation enables users to exert more precise control. However, this mode is less precise than the arm pose estimates in the Upper Arm mode. The Pocket mode, therefore, balances the precision and convenience of the other two modes.

### 4.2 Significance and limitations

WearMoCap enables motion capture from smartwatches and smartphones. Apart from the atmospheric pressure sensor and microphone data, collected measurements are identical to those provided by other IMU devices designed for motion capture purposes, e.g., Movella’s XSens Suite (Roetenberg et al., 2009). The significant difference between WearMoCap and established IMU solutions like XSens lies in the ubiquity and familiarity of smart devices for the average user. Smartphones and smartwatches are more widespread than customized IMU units, and a large population is familiar with starting and using apps on Android OS. While our motion capture methodology would perform equally well with customized IMUs (Prayudi and Kim, 2012; Beange et al., 2018; Li et al., 2021), it is the ubiquity of smart devices that makes WearMoCap attractive for future research into low-barrier robot control interfaces.

A limitation of WearMoCap is that, because of their reliance on IMUs, the global orientation estimates of smartwatches and smartphones can be subject to sensor drift. While the virtual orientation sensors of Android or Wear OS are robust to short-lived disturbances, e.g., moving a magnet past the device, slower long-term shifts can cause considerable offsets. The Android OS estimates device orientations through sensor fusion from accelerometer, magnetometer, and gyroscope using an Extended Kalman filter. Gyroscope drift is compensated by the gravity estimate from the accelerometer and the magnetic North from the magnetometer. As a result, the orientation is mostly subject to drift around the yaw axis due to shifts in the measured magnetic North. Our training and test data includes recording sessions of up to 10 min duration. Further, during the real-robot tasks, pose estimations typically stayed robust for 15 min or longer, but we had to ask subjects to recalibrate in about 10% or the instances. To mitigate sensor drift during longer sessions, a promising direction for future work involves utilizing our employed stochastic forward passes, which result in widening solution distributions when unrealistic changes or unergonomic angles occur (also depicted in Figure 5). This way of recognizing unergonomic or impossible angles from wide distributions can help mitigating sensor drift by automatically triggering recalibration.

Another source of drift is the sensor-to-segment misalignment, i.e., if the watch is loosely worn and slips post-calibration, we expect the tracking accuracy to be affected. In our experiments, we fitted the subjects with tightly strapped watches and phones to minimize this issue. However, in the future, we can look at better understanding the impact of sensor-to-segment misalignment and adopt techniques to correct it.

A further potential limitation common to phone-based apps is that major Operating System (OS) update, e.g., Android 12 to 13, could break our application if not updated properly to handle the OS change. However, some of our older tested devices, e.g., the OnePlus N100, do not receive long-term support anymore and will not undergo major updates in the future. It is unlikely WearMoCap will break on such older devices. Android OS updates for newer devices are rolled out slowly. To handle these updates in the long run, we have enabled the Issue Tracking function in the Github repository.

Another limitation is that our method assumes default arm lengths. While this is representative of the population that we tested with, unusually long or short arm lengths might adversely affect the tracking performance. Future work will investigate the effects of large variations in anthropometry. We publish WearMoCap as open source with this work to facilitate such future investigations. Lastly, we expect that we can improve the tracking performance by adding more subjects with varied motions and differing limb lengths.

### 4.3 Conclusion

This work presented WearMoCap, an extensively documented open-source library for ubiquitous motion capture and robot control from a smartwatch and smartphone. It features three motion capture modes: Watch Only requires the least setup; Upper Arm is the most precise; and Pocket is the most flexible. We benchmarked these modes on large-scale datasets collected from experiments with multiple human subjects and devices. To evaluate their practical use, we demonstrated and discussed their application in four real-robot tasks. Results show that, when chosen for the appropriate task, WearMoCap serves as an ubiquitous and viable alternative to the costly state-of-the-art motion capture systems. Future work involves evaluating the applicability of WearMoCap in more scenarios and implementing strategies for mitigating sensor drift. To this end, the WearMoCap library is published as open source together with step-by-step instructions and all training and test data.

## Data Availability

The raw data supporting the conclusions of this article will be made available by the authors, without undue reservation.

## Footnotes

1
https://cloud.google.com/speech-to-text
